# Investigation of Tool Wear and Chip Morphology in Dry Trochoidal Milling of Titanium Alloy Ti–6Al–4V

**DOI:** 10.3390/ma12121937

**Published:** 2019-06-16

**Authors:** Dongsheng Liu, Ying Zhang, Ming Luo, Dinghua Zhang

**Affiliations:** Key Laboratory of Contemporary Design and Integrated Manufacturing Technology of Ministry of Education, Northwestern Polytechnical University, Xi’an 710072, China; 2014200870@mail.nwpu.edu.cn (D.L.); zhangyingcdim@nwpu.edu.cn (Y.Z.); dhzhang@nwpu.edu.cn (D.Z.)

**Keywords:** tool wear, trochoidal milling, titanium alloy, chip morphology

## Abstract

Titanium alloys are widely used in the manufacture of aircraft and aeroengine components. However, tool wear is a serious concern in milling titanium alloys, which are known as hard-to-cut materials. Trochoidal milling is a promising technology for the high-efficiency machining of hard-to-cut materials. Aiming to realize green machining titanium alloy, this paper investigates the effects of undeformed chip thickness on tool wear and chip morphology in the dry trochoidal milling of titanium alloy Ti–6Al–4V. A tool wear model related to the radial depth of cut based on the volume of material removed (VMR) is established for trochoidal milling, and optimized cutting parameters in terms of cutting speed and axial depth of cut are selected to improve machining efficiency through reduced tool wear. The investigation enables the environmentally clean rough machining of Ti–6Al–4V.

## 1. Introduction

The application of titanium alloys has dramatically increased in many industries over the past half century, particularly in the aviation and aerospace industries, where about 80% of titanium production is used [1]. Such extensive application of titanium alloys is due to their excellent properties, such as high strength-to-weight ratio, good anticorrosion performance, and strong fracture resistance. Ti–6Al–4V accounts for the majority of the total consumption of all kinds of titanium alloys.

For most titanium alloy parts, especially aviation parts, traditional cutting methods such as turning, milling, drilling, and grinding are still the main means of processing and manufacturing [2,3]. However, titanium alloy is known as a kind of hard-to-cut material because of its several inherent properties, such as high chemical reactivity, low thermal conductivity, and low modulus of elasticity [4]. High chemical reactivity makes chip easily adhere to the tool-cutting edge. Low thermal conductivity increases the temperature at the tool cutting edge [5], which has an adverse effect on tool condition. Hence, tool wear is severe in the machining process of titanium alloy, and it will lead to serious machining vibration [6,7] or damage the machined surface quality [8]. In order to improve the tool life in the machining of titanium alloy, many research efforts have been made to assist in choosing suitable machining conditions. Effective cooling methods can significantly improve tool life by lowering the temperature of the tool cutting edge. Sun et al. [9] adopted the cryogenic compressed air cooling technique to cool the tool edge during the turning of Ti–6Al–4V. Pittalà [10] used CO_2_ cryogenic coolant in the end milling of Ti–6Al–4V. Bermingham et al. [11] made a comparison between cryogenic and high-pressure emulsion cooling technologies during the turning of Ti–6Al–4V, and found that high-pressure emulsion cooling achieved a slightly better tool life. Recently, minimum quantity lubrication (MQL) combined with cryogenic gas [12,13,14,15], such as carbon dioxide (CO_2_) and liquid nitrogen (LN2), has drawn great attention due to its excellent performance in improving tool life during machining hard-to-cut materials. Machining parameters such as the cutting speed, cutting depth, and feed rate have critical effects on tool life. Hou et al. [16] investigated the influence of cutting speed on tool wear in the end milling of Ti–6Al–4V, and the results showed the mean flank temperature and cutting force increased significantly as the cutting speed increased, which accelerated the tool wear. Jaffery et al. [17] indicated that the depth of cut had a significant effect on wear performance in machining Ti–6Al–4V alloy. To improve the machinability of Ti–6Al–4V, other approaches, such as laser-assisted machining [18], hybrid machining [19], and ultrasonic machining [20] were adopted by many researchers.

The improvement of tool life by the above-mentioned methods is at the expense of increased cost and energy consumption, even environmental pollution. To achieve the goals of green machining as well as the reduction of production cost, dry machining is considered as a promising and satisfactory solution in the future [21]. In the case of dry machining, the cleaning process of the workpiece can be spared, and the chips can be disposed of immediately without other treatment. Dry machining also has some positive effects, such as a reduction in thermal shock and no intrusion introduced by cooling equipment. However, the dry machining of titanium alloys is detrimental to the tool condition, because there will be more friction and adhesion between the tool and the workpiece without coolant, which will accelerate the tool wear [22]. Li et al. [23] investigated the effect of high cutting speed on tool wear in the dry milling of Ti–6Al–4V, and found that the increase in the cutting speed and feed rate accelerated the tool wear and drastically decreased the tool lifetime. Deng et al. [24] studied tool wear in the dry milling of Ti–6Al–4V, and found element diffusion from the Ti–6Al–4V titanium alloy to WC/Co carbide tools (and vice versa) at temperatures up to 400 °C. Li et al. [25] indicated that the main wear mechanisms of coated cemented carbide tools were the complicated interactions of several kinds of wear patterns—such as abrasive wear, coating delamination, adhesive wear, oxidation wear, and diffusion wear—at high cutting speed in the dry face milling of Ti–6Al–4V.

Trochoidal milling is a promising technology for the high-efficiency machining of hard-to-cut materials. This method is a combination of circular milling and slicing [26], which possesses a small engagement region between the tool and workpiece, and enables a relatively high feed rate and axial depth of cut to increase the machining efficiency. In trochoidal milling, the small engagement angle between the tool and workpiece generates low cutting forces, and a long period of non-engagement between the tool and workpiece ensures the effective cooling of the tool. These advantages enable a long tool life in trochoidal milling. Some research studies about trochoidal milling showed good practical results. Uhlmann et al. [27] indicated that trochoidal milling enabled lower energy consumption and process time in the machining of Ti–6Al–4V, compared with the conventional milling strategy. Wu et al. [28] investigated trochoidal milling in nickel-based superalloy, and the results showed that trochoidal milling significantly reduced tool flank wear. Patil et al. [29] adopted a trochoidal milling technique in the slot machining of Ti–6Al–4V, and found that it was better for surface finish and chip evacuation compared with the traditional slot milling method. In view of the merits of trochoidal milling, it is an effective method to reduce tool wear, as well as improve the machining efficiency and quality.

As mentioned above, dry trochoidal milling can be a potential high-efficiency green machining strategy. However, on reviewing the current research for the green milling of Titanium alloys, there appears to be limited reports on the machinability of the dry trochoidal milling of titanium alloy. To fill this research gap, this paper investigates the tool wear as well as the chip morphology in the dry trochoidal milling of titanium alloy Ti–6Al–4V based on a set of cutting experiments. Firstly, the effect that undeformed chip thickness had on tool wear and chip morphology was investigated in the dry milling of Ti–6Al–4V. The effects of radial cutting depth as well as cutting speed on tool wear were studied, and a tool wear model based on the volume of material removed (VMR) was then established based on the flank milling test. Finally, a set of optimized cutting parameters for the dry trochoidal milling of Ti–6Al–4V was selected and validated by cutting experiments.

## 2. Experimental Procedures

**Figure 1:**
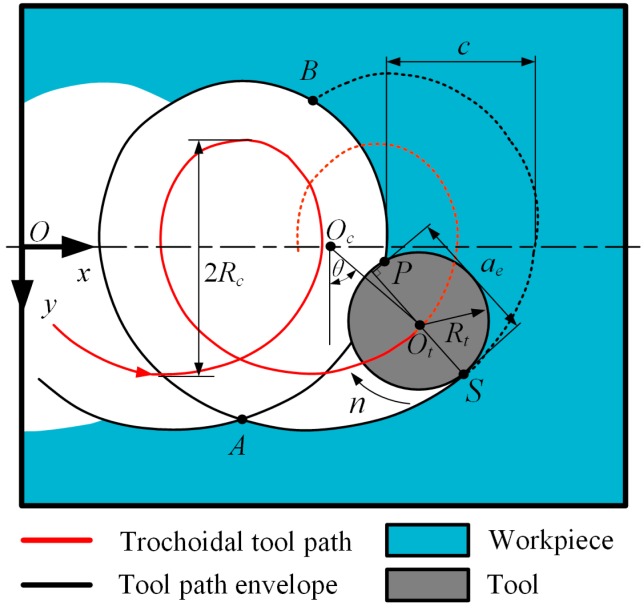
Geometric model of trochoidal milling. Here, *O_t_* is the tool center position; *O_c_* the current revolution center of the tool center; *θ* is the revolution angle; *c* is the trochoidal stepover; *R_c_* is the trochoidal radius; *R_t_* is the tool radius; and *a_e_* is the current radial depth of cut.

The trochoidal model enables continuity in both feed rate and acceleration due to its continuity in tangency and curvature [30], which is considered better concerning the kinematical behavior of the machine tool. The trochoidal model is shown in Figure 1. The tool center position *O_t_* (*x_c_*,*y_c_*) parameterized by angle *θ* is expressed as follows:

(1) $$\begin{array}{l} x_{c}=c\frac{\theta }{2\pi }+R_{c}\sin\theta \\ y_{c}=R_{c}\cos\theta \end{array}$$

where *c* is the trochoidal stepover, and *R_c_* is the trochoidal radius. Point *S* is on the tool path envelope, and can be calculated using:

(2) $$\vec{OS}=\vec{OO_{t}}+R_{t}\cdot \frac{\vec{O_{t}S}}{\left\|\vec{O_{t}S}\right\|}$$

where *R_t_* is the tool radius, and vector $\vec{O_{t}S}$ is normal to the trochoidal tool path. The tool contour (*x_t_*,*y_t_*) is determined by following expression:

(3) $${\left(x_{t}-c\frac{\theta }{2\pi }-R_{c}\sin\theta \right)}^{2}+{\left(y_{t}-R_{c}\cos\theta \right)}^{2}={R_{t}}^{2}$$

Point *P* is the intersection point of the tool contour and tool path envelope, and can be solved by Equations (2) and (3). After calculating *P*, the instantaneous radial depth of cut can be obtained using:

(4) $$a_{e}=\frac{\vec{PS}\cdot \vec{O_{t}S}}{\left\|\vec{O_{t}S}\right\|}$$

Point *A* and point *B* are the tool entry and exit points in one trochoidal cycle. Between *A* and *B*, the instantaneous radial depth of cut increases continuously until reaching its maximal value when point *P* reaches the cusp [31], and then decreases to zero. The trochoidal model generates a non-symmetric tool path that is smooth toward its cusp, which is not located on x axis.

To improve machining efficiency, trochoidal milling usually adopts a large axial depth of cut and a small radial depth of cut. In combination with suitable cutting parameters and machining equipment, an axial depth of cut up to two times the tool diameter can be reached [27]. Compared with the axial depth of cut, the radial depth of cut related to chip thickness has a greater effect on the tool wear, since it affects the maximum undeformed chip thickness, which determines the maximum cutting force applied on the tool cutting edge. Additionally, cutting speed also has a great effect on tool life in milling titanium alloy [16]. Therefore, before performing the dry trochoidal milling test, the effects of radial cutting depth and cutting speed on the dry milling of Ti–6Al–4V are investigated by flank milling tests first.

### 2.1. Experimental Setup

**Figure 2:**
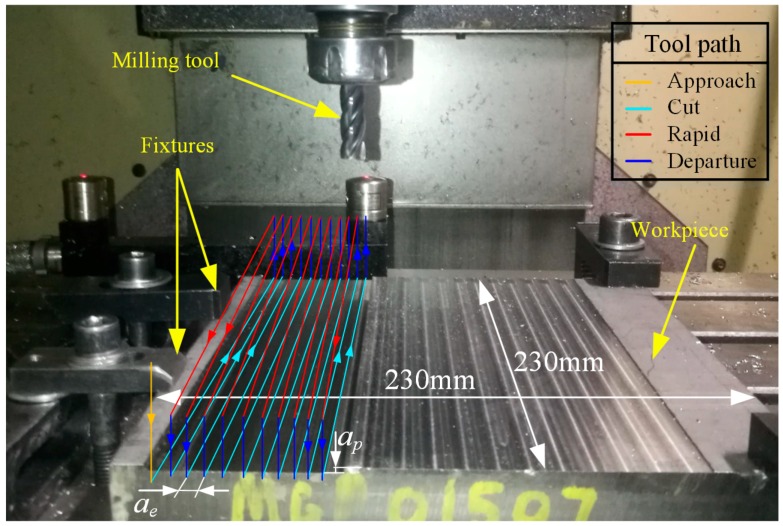
Experimental setup.

The workpieces used in this study were annealed Ti–6Al–4V blocks with a size of 230 mm × 230 mm × 27 mm. The nominal chemical components and physical properties of the titanium alloys are listed in Table 1. The specifications of the used cutting tools are presented in Table 2. All the milling tests were conducted on an YHV850 three-axis machine tool (Yonghua Machinery Co., Ltd., Yanzhou, China) shown in Figure 2. Down milling was adopted in all the tests.

### 2.2. Experimental Design

The experimental design is focused on two main cutting parameters: the cutting speed and the radial depth of cut. The axial depth of cut is fixed at 2 mm, and the feed rate is fixed at 0.1 mm per tooth. Three levels of cutting speeds are selected, i.e., *Vc* = 60 m/min, 130 m/min, and 200 m/min. For each cutting speed, there are three levels of radial depths of cut, i.e., *a_e_* = 0.2 mm, 0.4 mm, and 0.6 mm. Detailed cutting parameters for all nine groups of tests are listed in Table 3.

For each test, the tool rejection or failure is judged according to the following criteria:

(1) Average flank wear *VB* = 0.2 mm;
(2) Maximum flank wear *VB*_max = 0.3 mm;
(3) Excessive chipping/flaking or catastrophic failure.

The test will be stopped when any of the above criteria is reached. For the third criterion, the test result is invalid, and the test will be repeated until meeting one of the first two criteria. Then, the VMR will be calculated according to the cutting length.

## 3. Results and Analysis

### 3.1. Tool Wear Model Related with Radial Depth of Cut

Tool life results in terms of VMR are shown in Table 4, where the third column “effective cutting time” denotes the total time when the tool is engaged with the workpiece, and “MRR” represents the material removal rate. Suppose that the relationship between the radial depth of cut and VMR possesses a quadratic form as follows:

(5) $$VMR=A_{2}{a_{e}}^{2}+A_{1}a_{e}+A_{0}$$

where *A*_2_, *A*_1_, and *A*_0_ are unknown coefficients.

**Figure 3:**
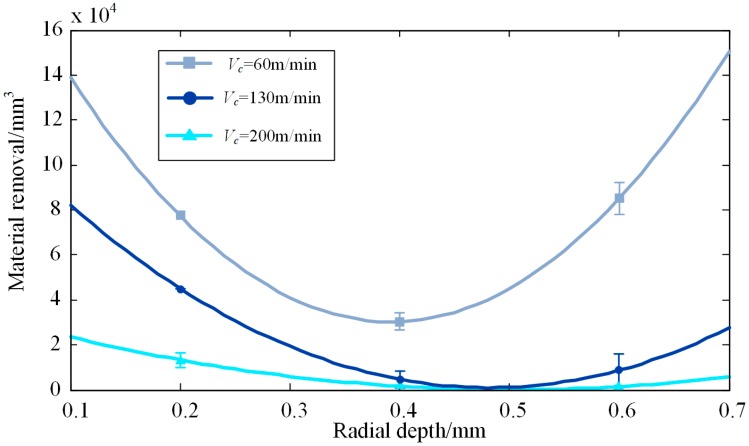
Relationship between radial depth of cut and VMR.

According to the results in Table 4, the relationships between radial depth of cut and VMR for three levels of cutting speeds can be obtained by quadratic fitting (see Figure 3):

(6) $$\begin{array}{l} VMR_{1}=1.2713\times 10^{6}{a_{e}}^{2}-0.9974\times 10^{6}a_{e}+0.2259\times 10^{5}\\ VMR_{2}=5.5603\times 10^{5}a_{e}-5.3556\times 10^{5}a_{e}+1.2995\times 10^{5}\\ VMR_{3}=1.4433\times 10^{5}{a_{e}}^{2}-1.4502\times 10^{5}a_{e}+0.3657\times 10^{5} \end{array}$$

where *VMR*_1_, *VMR*_2_, and *VMR*_3_ correspond to cutting speeds of 60 m/min, 130 m/min, and 200 m/min, respectively. In Figure 3, it is observed that VMR decreases with the increase of cutting speed in the cutting speed range of 60 to 200 m/min. For each cutting speed, there is a radial depth of cut corresponding to the lowest VMR, which should be avoided in practical milling. More specifically, VMR at 0.4-mm radial depth of cut is lower than that at 0.2 mm and 0.6 mm radial depths of cut. This could be explained by the cutting force load increasing with the increase of radial depth of cut, thus accelerating the tool wear. However, when the radial depth of cut increased to 0.6 mm, a thicker chip carried away more cutting heat, which kept the tool in a better cutting condition despite a larger force load.

### 3.2. Tool Wear and Chip Morphology

**Figure 4:**
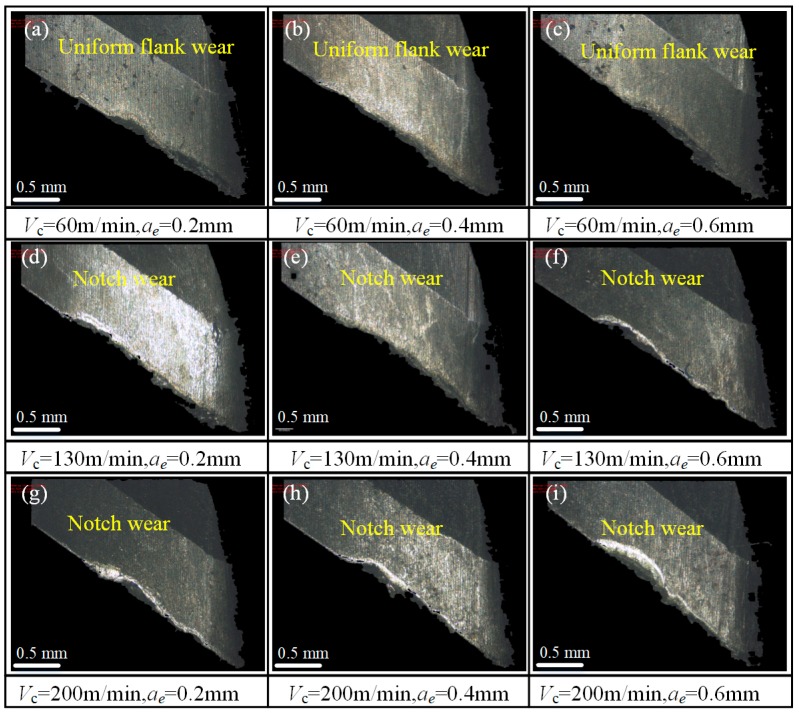
Photos taken by Alicona Infinitefocus G4 for worn tools with cutting parameters (**a**) *V*_c_ = 60 m/min, *a*_e_ = 0.2 mm, (**b**) *V*_c_ = 60 m/min, *a*_e_ = 0.4 mm, (**c**) *V*_c_ = 60 m/min, *a*_e_ = 0.6 mm, (**d**) *V*_c_ = 130 m/min, *a*_e_ = 0.2 mm, (**e**) *V*_c_ = 130 m/min, *a*_e_ = 0.4 mm, (**f**) *V*_c_ = 130 m/min, *a*_e_ = 0.6 mm, (**g**) *V*_c_ = 200 m/min, *a*_e_ = 0.2 mm, (**h**) *V*_c_ = 200 m/min, *a*_e_ = 0.4 mm, and (**i**) *V*_c_ = 200 m/min, *a*_e_ = 0.6 mm.

Tool edges of worn tools were scanned using Alicona Infinitefocus G4 (Graz, Austria) with a magnification of 50×, as shown in Figure 4. For three groups of tests at 60 m/min cutting speed (test no. 1, 2, and 3), the tool wear pattern is uniform flank wear. As the cutting speed increases to 130 m/min and 200 m/min, the tool wear pattern changes to notch wear, and the maximum wear position locates on the upper part of the tool edge. At the same radial depth of cut, the maximum flank wear increases as the cutting speed increases. At the same cutting speed, the higher the radial depth, the more serious the tool wear. For all the tests, tool wear on the tool tip is slight. As reported in [9], in milling titanium alloy, the cutting temperature increases dramatically with the increase in the cutting speed, and the tool wear progresses rapidly due to the high temperature and strong adhesion between the tool and workpiece material.

**Figure 5:**
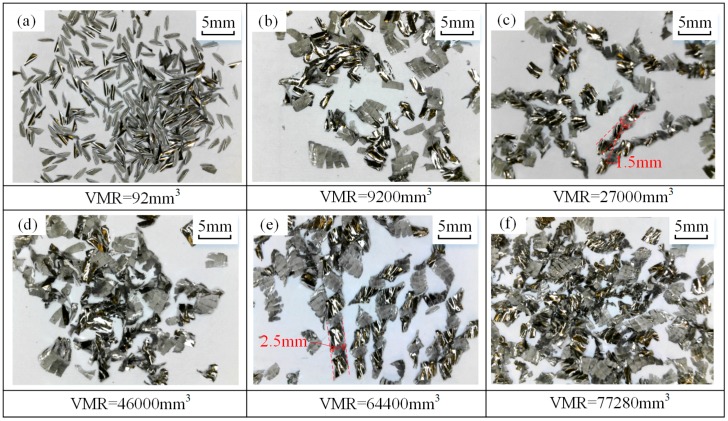
Chip morphology (test no. 1: *V_c_* = 60 m/min, *a_p_* = 2 mm, *a_e_* = 0.2 mm, *f_z_* = 0.1 mm/tooth) under VMR of (**a**) 92 mm^3^, (**b**) 9200 mm^3^, (**c**) 27,000 mm^3^, (**d**) 46,000 mm^3^, (**e**) 64,400 mm^3^, and (**f**) 77,280 mm^3^.

**Figure 6:**
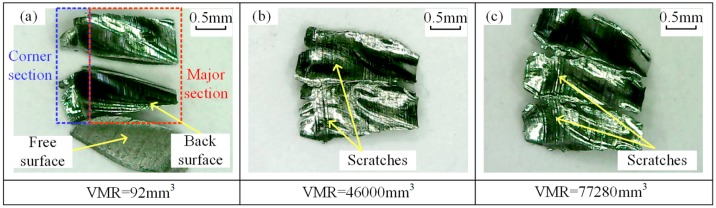
Chip morphology in enlarged view (test no. 1: *V_c_* = 60 m/min, *a_p_* = 2 mm, *a_e_* = 0.2 mm, *f_z_* = 0.1 mm/tooth) with VMR of (**a**) 92 mm^3^, (**b**) 46,000 mm^3^, and (**c**) 77,280 mm^3^.

Chips during tests were collected and analyzed. The macro images of chips in test no. 1 are shown in Figure 5, where chip morphologies at different VMRs are presented. At the initial stage of tool life, the chips are uniform, regular, curled, and separated in shape due to intermittent milling and good tool condition [32] (see Figure 5a). Each chip can be divided into two sections: the corner section and the major section (see Figure 6a). The corner section was formed by the tool nose edge, and the major section was formed by the side cutting edge [33]. The back surface of the chip came into contact with the rake face of the tool under high contact pressure, and the friction is smooth and shiny, while the free surface of the chip is rough (see Figure 6a). When the VMR reaches 9200 mm^3^, chip separation has failed, which results in several chips sticking to each other (see Figure 5b). With the VMR increased to 27,000 mm^3^, chips with tight spiral shapes appear (see Figure 5c). As the VMR increases, the spiral radius increases (see Figure 5c,e). When the VMR is 46,000 mm^3^, the chip is twisted and irregular in shape. Besides, obvious scratches related to tool wear are observed on the back surface of the chip (see Figure 6b). At the end stage of tool life, serious distortion and scratches occur on the chip (see Figure 6c). It can be inferred that the worse the tool wear, the more severe the chip distortion.

**Figure 7:**
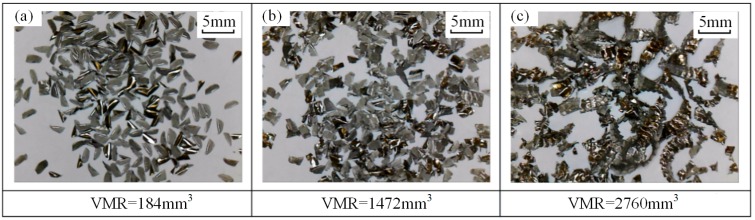
Chips morphology (test no. 5: *V_c_* = 130 m/min, *a_p_* = 2 mm, *a_e_* = 0.4 mm, *f_z_* = 0.1 mm/tooth) under VMR of (**a**) 184 mm^3^, (**b**) 1472 mm^3^, and (**c**) 2760 mm^3^.

**Figure 8:**
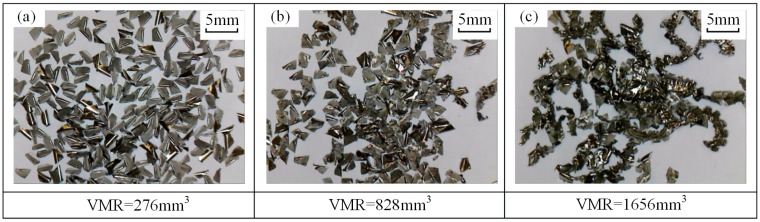
Chips morphology (test no. 9: *V_c_* = 200 m/min, *a_p_* = 2 mm, ae = 0.6 mm, *f_z_* = 0.1 mm/tooth) under VMR of (**a**) 276 mm^3^, (**b**) 828 mm^3^, and (**c**) 1656 mm^3^.

**Figure 9:**
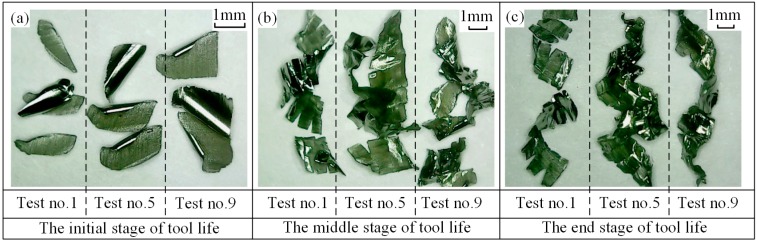
Chip morphology at (**a**) initial stage (the VMR values for test no. 1, no. 5, and no. 9 are 92 mm^3^, 184 mm^3^, and 276 mm^3^), (**b**) middle stage (the VMR values at the middle stage for test no. 1, no. 5, and no. 9 are 46,000 mm^3^, 1472 mm^3^, and 828 mm^3^) and (**c**) end stage (the VMR values at the end stage for test no. 1, no. 5, and no. 9 are 77,280 mm^3^, 2760 mm^3^, and 1656 mm^3^) of tool life.

Chip morphologies in tests no. 5 and no. 9 are shown in Figure 7 and Figure 8, respectively. Comparisons of chip morphology between test no. 1, no. 5, and no. 9 are shown in Figure 9. The evolutions of chip morphology are similar for each test. At the initial stage of tool life, the chips are separated and regular in shape (see Figure 7a, Figure 8a and Figure 9a). At the middle stage, the chips begin to stick together (see Figure 7b, Figure 8b and Figure 9b). At the end stage, continuous chips with long spiral shapes appear (see Figure 7c, Figure 8c and Figure 9c), while the chips in test no. 1 are broken into pieces. Such a difference of chip morphologies between test no. 1 and test no. 5 or no. 9 is caused by the chip thickness. To be more specific, the radial depth of cut in test no. 1 is smaller than those in test no. 5 and no. 9, which causes the chip in test no. 1 to be thinner and easier to break. Chip morphologies in other tests were also observed, and it was found that the evolutions of chip morphology at the same radial depth were similar. The change of chip morphology is mainly caused by the tool wear and the formation of a built-up edge [34].

The results show that the cutting speed and the radial depth of cut have great influence on the VMR. To achieve a maximum VMR, the suggested cutting parameters are *V_c_* = 60 m/min and *a_e_* = 0.6 mm when *a_p_* = 2 mm and *f_z_* = 0.1 mm/tooth in drying milling Ti–6Al–4V.

### 3.3. Trochoidal Milling Test

**Figure 10:**
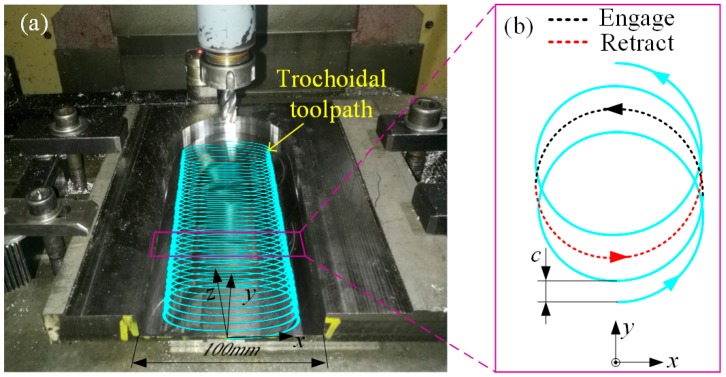
(**a**) Dry trochoidal milling test; (**b**) Trochoidal toolpath.

The test setup for trochoidal milling is shown in Figure 10a. The tool has the same specifications as those used in the above tests. Figure 10b shows the trochoidal toolpath, where c denotes the trochoidal stepover. One trochoid cycle is composed of a toolpath of retraction denoted by the red dot line and a toolpath of engagement denoted by the black dot line (see Figure 10b). According to the results of the flank milling tests, a combination of a 60 m/min cutting speed and 0.6-mm radial depth of cut achieved a maximum VMR. Thus, a set of cutting parameters—i.e., *V_c_* = 60 m/min, *f_z_* = 0.1 mm/tooth, and *c* = 0.6 mm—was adopted in the trochoidal milling test, where the stepover corresponded to the radial depth of cut. Unlike the above tests, the axial depth of cut was set to 10 mm in this test to achieve a high material removal rate.

**Figure 11:**
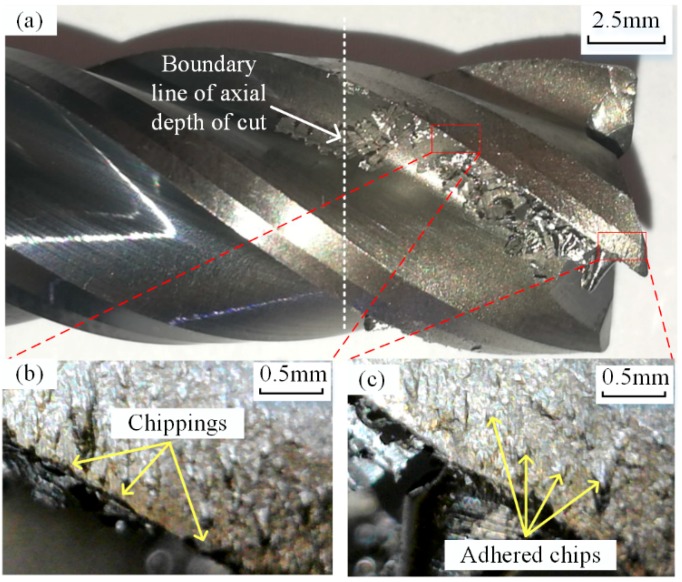
Tool condition after dry trochoidal milling test. (**a**) General view of the tool; (**b**) Top position; (**c**) Middle position; (**d**) Bottom positon.

This test achieved a material removal rate of 1165.9 mm^3^/min and a total volume of 206,940 mm^3^ of material removed. The total cutting time, including the tool engaged and retracted time, was 177.5 min. The general view of the tool after the test is shown in Figure 11a, where lots of chip debris adhered to the rake face of the tool are observed, and the shapes of chips are diverse along the cutting edge. In addition, the TiAlN coating (dark color) on the engaged part of the tool had been worn away and the substrate material (bright color) of the tool was exposed. Figure 11b,c show enlarged views of the middle and tip position of the engaged cutting edge, respectively. In the figures, the materials removed from the workpiece were adhered to the flank face by cold welding. Moreover, small chippings, which perform as chip breakers, can be observed on the cutting edge [35].

**Figure 12:**
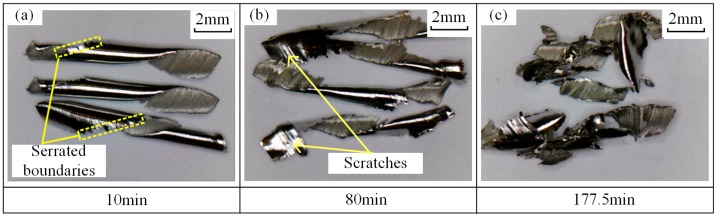
Chip morphology in trochoidal milling tests at cutting stage of (**a**) 10 min, (**b**) 80 min and (**c**) 177.5 min.

The chips at different stages of tool life were collected in the trochoidal milling tests. Chip morphology images are shown in Figure 12. It is observed that each chip had an intact curled needle shape with serrated boundaries at an early stage of 10 min (see Figure 12a). At 80 min, distorted and obvious scratches could be seen on the back surface of the chip (see Figure 12b). At 177.5 min, several chips were twisted into a lump, and intact chips had broken into small pieces (see Figure 12c). It can be concluded that chip morphology is closely related to the tool wear condition and provides an important indication for tool condition monitoring.

## 4. Conclusions

This paper investigated the tool wear and chip morphology in the dry milling of titanium alloy Ti–6Al–4V for the aim of green machining titanium alloy. The effect of the radial depth of cut on tool wear was studied considering cutting speed, and a corresponding tool wear model related with radial cutting depth was established. According to the test results, cutting parameters in terms of radial depth of cut were optimized to improve the machining efficiency and reduce the tool wear. Then, the optimized cutting parameters were adopted by following the trochoidal milling test. Meanwhile, chips over the tool life cycle were collected and observed. Based on the results and analysis, the following conclusions can be drawn:

(1) For each cutting speed, *V_c_* = 60, 130, and 200 m/min, there is a radial depth of cut corresponding to the lowest VMR, which should be avoided in practical milling. In this research, a 0.4-mm radial depth of cut should be avoided.

(2) In order to improve cutting efficiency in material removal, an appropriate radial depth of cut is necessary. In this paper, a 0.6-mm radial depth of cut combined with a 60 m/min cutting speed are suggested when *a_p_* = 2 mm and *f_z_* = 0.1 mm/tooth in drying milling titanium alloy Ti–6Al–4V.

(3) Trochoidal milling is a promising method for green machining titanium alloy. By using a 0.6-mm radial depth of cut and 60 m/min cutting speed, the material removal rate is 1165.93 mm^3^/min, and the tool life reaches 177.5 min in dry trochoidal milling.

(4) Chip morphology is closely associated with tool condition, and chip morphology can be adopted to monitor the tool condition.

## Figures and Tables

**Table 1:** Nominal chemical components and physical properties of the titanium alloys.

Element	Al	V	Fe	O	C	N	H	Ti
wt.%	5.5–6.8	3.5–4.5	<0.5	<0.2	<0.1	<0.05	<0.015	balance
**Physical Properties**	**Density** **(kg/m^3^)**	**Elastic** **Modulus (GPa)**	**Yield Strength** **(MPa)**		**Thermal Conductivity** **(W/(m·K))**		**Hardness** **(HRC)**	**Melting Point** **(°C)**
value	4430	113.8	880		6.7		36	1604–1660

**Table 2:** Specifications of the helical milling cutter.

Diameter (mm)	Number of Flutes	Helix Angle (°)	Corner Radius (mm)	Coating	Rake Angle (°)	Clearance Angle (°)	Second Clearance Angle (°)	Hardness (HRC)
12	4	35	0.5	TiAlN (3 μm)	4	8	20	65

**Table 3:** Cutting parameters used in the experiments (*f_z_* = 0.1 mm/tooth, *a_p_* = 2 mm).

Test No.	Cutting Speed (m/min)	Radial Depth of Cut *a_e_* (mm)	Spindle Speed (rpm)	Feed Rate (mm/min)
1	60	0.2	1592	637
2		0.4		
3		0.6		
4	130	0.2	3448	1379
5		0.4		
6		0.6		
7	200	0.2	5305	2122
8		0.4		
9		0.6		

**Table 4:** Test results. VMR: volume of material removed.

Test No.	VMR (mm^3^)	Effective Cutting Time (s)	MRR (mm^3^/min)
1	77,280	18,198	254.8
2	30,360	3575	509.6
3	85,146	6683	764.4
4	45,080	4904	551.6
5	4692	255	1103.2
6	8786	319	1654.8
7	13,340	943	848.8
8	1472	52	1697.6
9	1518	36	2546.4
